# Correlates of anxiety and depressive symptoms in inpatients with COVID-19 in Taiwan

**DOI:** 10.1016/j.heliyon.2023.e20679

**Published:** 2023-10-05

**Authors:** Wei-Chen Lee, Chun-Lin Chen, Yi-Ju Pan

**Affiliations:** a Department of Psychiatry, Far Eastern Memorial Hospital, New Taipei City, Taiwan; b Center for General Education, Taipei Medical University, Taipei, Taiwan; c Department of Chemical Engineering and Materials Science, Yuan Ze University, Taoyuan City, Taiwan

**Keywords:** COVID-19, Anxiety, Depression, Hospitalization, Hospital anxiety and depression scale (HADS)

## Abstract

**Objectives:**

Inpatients with COVID-19 may experience high levels of anxiety and depressive symptoms during the pandemic. No prior study has examined these symptoms with COVID-19 inpatients in Taiwan. Using data from a tertiary hospital in Northern Taiwan, we investigated anxiety and depressive symptoms and the associated sociodemographic or clinical characteristics in these patients.

**Methods:**

Data of anxiety and depressive symptoms by the Hospital Anxiety and Depression Scale (HADS) as well as the sociodemographic and clinical correlates were retrospectively retrieved and analyzed for COVID-19 patients admitted to Far Eastern Memorial Hospital from June 4 to June 28, 2021.

**Results:**

In total, 152 patients with COVID-19 were included. Among all the COVID-19 inpatients, 9.9 % (n = 15) had an HADS anxiety score of ≥8 and 7.2 % (n = 11) had an HADS depression score of ≥8. COVID-19 inpatients with HADS anxiety score ≥8 or HADS depression score ≥8 were found to have a longer length of hospital stay compared to the respective comparison group. The female patients, patients aged >55 years, and patients hospitalized for >15 days had significantly higher anxiety scores than did the corresponding comparison groups.

**Conclusion:**

COVID-19 inpatients with either anxiety or depression were associated with longer length of hospital stay. Age, sex, and hospitalization length were found to be associated with anxiety symptoms in inpatients with COVID-19. Future studies are warranted to elucidate differential mechanisms potentially related to anxiety and depressive symptoms in patients with COVID-19.

## 1 Introduction

The COVID-19 outbreak in late 2019 has been sweeping the world since 2020. COVID-19 infection was initially known to directly caused pulmonary damage [1]. However, with an increasing understanding of the virus, extrapulmonary involvements have been noted in many systems, such as cardiac, thromboembolic, and neurologic manifestations [2]. As COVID-19 continues to spread, people are becoming more aware of its impact on mental health. Measures like isolation, lockdown, and social distancing, implemented globally to block the COVID-19 transmission, have led to challenges such as financial insecurity, unemployment, loneliness, work pressure, and uncertainty about the future [[3], [4], [5]]. These extended effects have triggered public emotional crisis [6,7]. Patients who had psychiatric disorders showed an increased risk of detrimental psychological outcomes during a strict COVID-19 lockdown [8]. Besides, anxiety, depression, worry, and loneliness in individuals without a history of psychiatric disorders also significantly deteriorated during the COVID-19 pandemic [9].

Patients with confirmed COVID-19 face stricter isolation measures and heightened concerns about their health and life. Moreover, COVID-19 can directly affect the brain, leading to the development of neuropsychiatric symptoms [10]. In this regard, research into the psychological states and neuropsychiatric symptoms of patients with COVID-19 is essential. Indeed, prior studies have reported that patients with COVID-19 felt lonely, anxious, or depressed [11,12]. Beyond anxiety and depressive symptoms, one meta-analysis revealed that COVID-19 patients had high rates of post-traumatic stress disorder, insomnia, somatization and fear [13]. However, compared with studies on the mental health of the general population, COVID-19 survivors, or healthcare personnel during COVID-19 pandemic, relatively few studies have investigated the mental health of inpatients with COVID-19 during their stay [[14], [15], [16], [17], [18], [19], [20]]. Moreover, the existing results with COVID-19 inpatients varied widely, with the reported prevalence of anxiety and depression ranging from 19 % to 60 % and from 13 % to 80 %, respectively [[15], [16], [17],^21^,^22^].

In Taiwan, no prior study has specifically evaluated anxiety and depressive symptoms in inpatients with COVID-19. The current study aimed to examine anxiety, depressive symptoms, and the related sociodemographic and clinical factors in COVID-19 inpatients in Taiwan.

## 2 Materials and methods

### 2.1 Study design and population

After the COVID-19 outbreak in May 2021, New Taipei City, the most populous administrative region in Taiwan (population: 4 million) had the highest number of confirmed COVID-19 cases; meanwhile, Far Eastern Memorial Hospital (FEMH) as the only medical center in Taiwan's New Taipei City, became one of the largest designated hospitals for inpatients with COVID-19. During the 2021 COVID-19 pandemic in Taiwan, FEMH treated approximately 11 % of patients with COVID-19 requiring intensive care in Taiwan [23].

The current study used the existing data based on electronic medical records and a telephone counseling on anxiety and depressive symptoms of COVID-19 inpatients. In line with the government's policy during the pandemic, the telephone counseling was to understand the mental health needs and to give emotional support to COVID-19 inpatients during their hospital stay and to provide further counseling sessions when it is needed. First, mental health professionals (psychologists or social workers) asked the in-charge nurses whether the patient was willing to provide his/her phone number and receive the telephone counseling call and only when it was agreed, the mental health professionals would make the calls. Therefore, patients who were not clear in consciousness, physically infeasible, or not able to give oral consent or receive telephone call (for example, on ventilator support), were excluded. At the beginning of the telephone calls, the mental health professionals would make a brief introduction of themselves and seek consent from the patients for the coming conversation. In total, the mental health professionals made calls to 165 COVID-19 inpatients and 152 agreed to talk to the professionals and answer the questionnaire via telephone. Among the 13 failed calls, 4 patients refused to receive the telephone counseling, 5 patients could not complete the calls because of physical conditions (n = 2), hearing impairment (n = 1), and cognitive function impairments (n = 2), and 4 patients provided wrong numbers or could not receive the calls during hospitalization. Among the 152 COVID-19 inpatients who completed the telephone counseling, 16 inpatients expressed their needs for further counseling and received additional counseling sessions via telephones during their hospital stay. The current study was based on data from the telephone counseling and electronic medical records. The study procedures were approved by the FEMH Research Ethics Committee (110120-E). The written informed consents were waived due to the retrospective design and the de-identified nature of the data analysis in the study.

Data of the individuals who were aged ≥15 years, diagnosed as having COVID-19 on the basis of a positive result for the SARS-CoV-2 polymerase chain reaction, admitted to FEMH from June 4 to June 28, 2021, and received the telephone counseling call, were included. This time frame matched the first peak of the 2021 COVID-19 pandemic in Taiwan. The patients’ sociodemographic and clinical characteristics were extracted from electronic medical records. Sociodemographic data included age, sex, educational level, marital status, living status (living alone or not), and smoking habit. Clinical data included body mass index (BMI), history of comorbidities associated with a higher risk of severe COVID-19 infection (including diabetes mellitus, hypertension, cerebrovascular disease, cardiovascular disease, pulmonary disease, and cancer) [24], history of psychiatric disorders (including schizophrenia spectrum and other psychotic disorders, bipolar disorders, depressive disorders, anxiety disorders, and insomnia), history of psychotropic medication use (including antipsychotics, mood stabilizers, antidepressants, and sedative-hypnotics), length of hospital stay, use of intubation, intensive care unit (ICU) stay, and the highest serum level of C-reactive protein (CRP)—a systemic inflammation marker (≥7.5 mg/dL, <7.5 mg/dL). Additionally, data regarding the use of specific COVID-19 treatments, including steroids, remdesivir, and monoclonal antibodies (e.g., Tocilizumab) were also extracted.

### 2.2 Measures

The anxiety and depressive symptoms of COVID-19 inpatients were measured using the Chinese version of the Hospital Anxiety and Depression Scale (HADS), a 14-item questionnaire. The HADS was developed by Zigmond and Snaith in 1983 for clinical patients and includes seven questions for assessing anxiety and depressive symptoms, respectively. The HADS adopts a 4-point Likert-type scale (0–3 points). Anxiety and depression are scored separately, with the total score being between 0 and 21. The higher the total score is, the higher the degree of anxiety or depression is. A score of <8 indicates no anxiety or depression, whereas a score of ≥8 is highly associated with anxiety or depression [25,26]. The HADS was validated in various populations with satisfactory internal consistency and concurrent validity [27,28]. The Chinese version of the HADS was validated in several prior studies [[29], [30], [31]].

### 2.3 Statistical analysis

Sociodemographic data (age, sex, educational level, marital status, living status, and smoking habit), clinical data (BMI, physical comorbidities associated with a higher risk of severe COVID-19 infection, history of psychotropic medication use, hospitalization length in days, ICU stay, use of intubation, and elevated CRP [highest CRP]), and data regarding the use of steroids, remdesivir, and tocilizumab during hospitalization were obtained for the overall sample and compared between groups based on the presence of anxiety (defined as the HADS cutoff score of ≥8) and depression (defined as the HADS cutoff score of ≥8), respectively. Continuous variables were compared using the two-sample *t*-test, and categorical variables were compared using the chi-squared test. Univariable analysis was conducted using the two-sample *t*-test and analysis of variance to examine whether and how these sociodemographic and clinical correlates affect HADS anxiety and depressive symptom scores, respectively. Separate multivariable linear regression analysis was performed to estimate the differential associations of sociodemographic and clinical characteristics with both the scores. The detailed exposure variables for multivariable regression analyses are presented in Table 3, Table 4. We further explored the relationship between presence of anxiety and depression symptoms and sociodemographic and clinical variables using Poisson regression models. The dependent variables, inpatients with HADS anxiety or depression score ≥8, followed Poisson distribution according to the Kolmogorov-Smirnov test. The results were presented in Table 5. All statistical analyses were performed using SPSS (version 25.0; IBM, Armonk, NY, USA), and the significance level was set at 0.05 (two-tailed) with a 95 % confidence interval.

**Table 1:** Demographic and clinical correlates by groups based on the HADS scores.

		Anxiety			Depression		
	Total (*n* = 152)	HADS-Anxiety<8 (*n* = 137)	HADS-Anxiety≥8 (*n* = 15)	*p*	HADS-Depression<8 (*n* = 141)	HADS-Depression≥8 (*n* = 11)	*p*
**Female gender, n (%)**	75 (49.3)	66 (48.2)	9 (60)	0.384	70 (49.6)	5 (45.5)	0.789
**Age [Mean (SD)]**	54.72 (14.25)	54.79 (14.27)	54.07 (14.49)	0.924	54.50 (14.52)	57.45 (10.21)	0.053
**Age [Median (IDR)]**	58 (21.75)						
**Age >55 y/o, n (%)**	90 (59.2)	81 (59.1)	9 (60)	0.948	82 (58.2)	8 (72.7)	0.344
**Educational status, n (%)**							
*Elementary school and below*	40 (26.3)	36 (26.3)	4 (26.7)	0.870	37 (26.2)	3 (27.3)	0.957
*High school*	79 (52)	72 (52.5)	7 (46.7)		73 (51.8)	6 (54.5)	
*University and above*	33 (21.7)	29 (21.2)	4 (26.7)		31 (22.0)	2 (18.2)	
**Not married, n (%)**	42 (27.6)	37 (27.0)	5 (33.3)	0.603	38 (27.0)	4 (36.4)	0.501
**Live alone, n (%)**	9 (5.9)	9 (6.6)	0 (0)	0.306	9 (6.4)	0 (0)	0.388
**Smoking, n(%)**							
*Non-smoker*	121 (79.6)	108 (78.8)	13 (86.7)	0.392	114 (80.9)	7 (63.6)	0.170
*Quit*	16 (10.5)	14 (10.2)	2 (13.3)		13 (9.2)	3 (27.3)	
*Current smoker*	15 (9.9)	15 (10.9)	0 (0)		14 (9.9)	1 (9.1)	
**BMI≥25, n(%)**	68 (45.9)	62 (45.3)	6 (40)	0.807	66 (46.8)	2 (18.2)	0.055
**History of psychiatric disorder diagnoses, n(%)**	10 (6.6)	9 (6.6)	1 (6.7)	0.988	10 (7.1)	0 (0)	0.361
**History of psychotropic medication use, n(%)**	11 (7.2)	10 (7.3)	1 (6.7)	0.928	10 (7.1)	1 (9.1)	0.805
**Comorbid physical illnesses related to severe COVID-19**^a^**, n(%)**	91 (59.9)	84 (61.3)	7 (46.7)	0.272	82 (58.2)	9 (81.8)	0.123
**Length of hospital stay in days [Mean (SD)]**	15.73 (8.91)	15.31 (8.24)	19.60 (13.33)	0.023*	15.12 (7.92)	23.55 (15.74)	0.001*
**Length of hospital stay in days [Median (IDR)]**	14.00 (6.00)						
**Length of hospital stay (>15 days), n(%)**	55 (36.2)	49 (35.8)	6 (40)	0.746	48 (34.0)	7 (63.6)	0.049*
**ICU stay, n(%)**	35 (23.0)	32 (23.4)	3 (20)	0.769	33 (23.4)	2 (18.2)	0.692
**Use of intubation**^b^**, n(%)**	13 (8.6)	11 (8.0)	2 (13.3)	0.486	12 (8.5)	1 (9.1)	0.947
**CRP≥7.5 mg/dl, n(%)**	63 (41.4)	57 (41.6)	6 (40)	0.905	56 (39.7)	7 (63.6)	0.121
**Use of steroid**^c^**, n(%)**	121 (79.6)	108 (78.8)	13 (86.7)	0.475	112 (79.4)	9 (81.8)	0.850
**Use of Remdesivir**^d^**, n(%)**	84 (55.3)	75 (54.7)	9 (60)	0.698	75 (53.2)	9 (81.8)	0.066
**Use of Tocilizumab**^e^**, n(%)**	62 (40.8)	58 (42.3)	4 (26.7)	0.241	56 (39.7)	6 (54.5)	0.335

Continuous variables were compared using a two-sample *t*-test; categorical variables were compared using a chi-squared test.

*p < 0.05.

Abbreviation: SD: Standard Deviation; BMI: Body Mass Index; ICU: Intensive Care Unit; CRP:C- Reactive Protein.

a Comorbid physical illnesses related to severe COVID-19 infection included diabetes mellitus, hypertension, cerebrovascular disease, cardiovascular disease, pulmonary disease, and cancer.

b Intubation: patients receiving intubation during hospitalization.

c Use of steroid: patients receiving steroids during hospitalization.

d Use of Remdesivir: patients receiving Remdesivir during hospitalization.

e Use of Tocilizumab: patients receiving Tocilizumab during hospitalization.

## 3 Results

### 3.1 Characteristics

In total, data of 152 hospitalized individuals with COVID-19 were included in the current analysis. As presented in Table 1, the female patients constituted 49.3 % of the study participants (mean age = 54.72 years; SD = 14.25 years). Most of them had high school education (52.0 %), were married (72.4 %), and lived with family members or others (94.1 %). In total, 9.9 % of the patients were current smokers, and a pre-obesity BMI of ≥25 was noted in 45.9 % of the inpatients [32]. Moreover, 6.6 % of the patients had a history of a psychiatric disorder, 7.2 % had a history of using psychotropic medications, and 59.9 % had physical comorbidities related to greater risk of developing severe COVID-19 infection, including diabetes mellitus, hypertension, cerebrovascular disease, cardiovascular disease, pulmonary disease, and cancer. The mean length of hospitalization was 15.73 (SD = 8.91) days, and 36.2 % of the patients were hospitalized for more than 15 days. In the study sample, 23 % of the patients were ever admitted to ICU during this hospital stay, and 8.6 % were intubated. Besides, 41.4 % of the patients exhibited the highest CRP level of ≥7.5 mg/dL. The patients receiving steroids, remdesivir, and tocilizumab comprised 79.6 %, 55.3 %, and 40.8 % of our study sample, respectively. The mean sample scores for anxiety and depression in the HADS were 3.76 (SD = 3.2) and 2.31 (SD = 2.9), respectively. The median sample scores for anxiety and depression in the HADS were 3 (IDR = 5) and 1 (IDR = 3), respectively.

### 3.2 HADS score ≥8

**Fig. 1:**
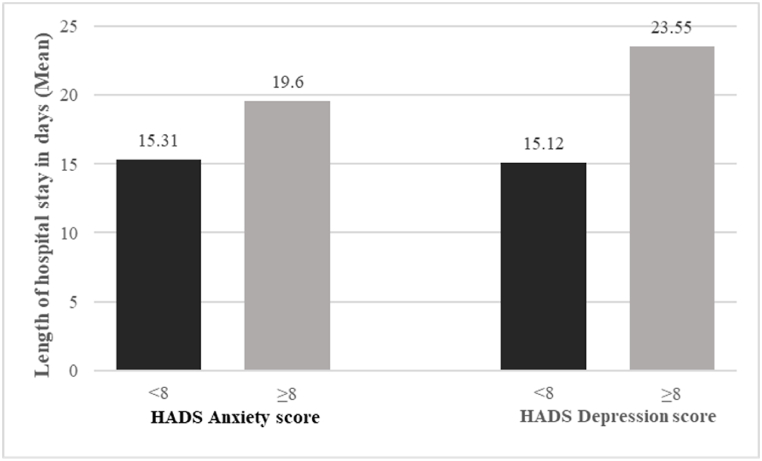
COVID-19 inpatients with HADS anxiety score ≥8 or HADS depression score ≥8 were found to have longer length of hospital stay. p < 0.05.

Using an HADS score of ≥8 as a cutoff point, we noted that 9.9 % and 7.2 % of the recruited inpatients with COVID-19 had anxiety and depressive symptoms. Six COVID-19 inpatients (3.9 %) were with HADS score ≥8 for both anxiety and depressive symptoms. Individuals with HADS anxiety score ≥8 were associated with longer length of hospital stay than did the corresponding comparison group. Those with HADS depression score ≥8 had longer length of hospital stay as well (Fig. 1).

**Fig. 2:**
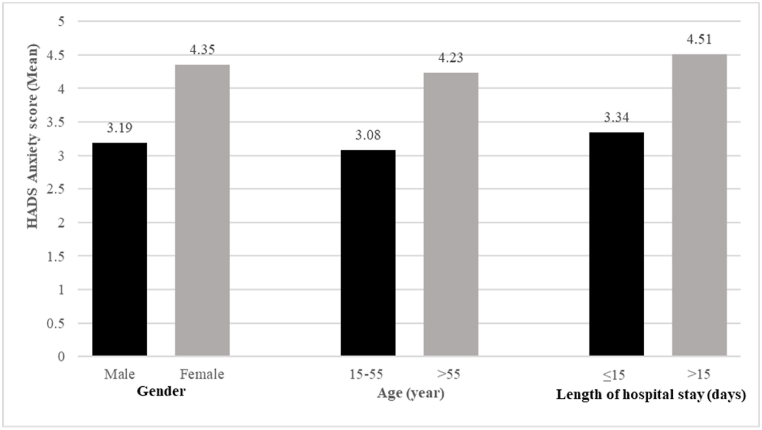
Female patients, patients aged >55 years, and patients hospitalized for more than 15 days had higher HADS anxiety scores. p < 0.05.

**Table 2:** Associations between HADS scores and demographic/clinical correlates among inpatients with COVID-19.

		HADS-Anxiety [Mean (SD)]	*p*	HADS-Depression [Mean (SD)]	*p*
**Age(yr)**	*15–55*	3.08 (3.20)	0.030*	1.97 (2.30)	0.230
	*>55*	4.23 (3.17)		2.54 (3.24)	
**Gender**	*Male*	3.19 (3.17)	0.027*	2.49 (3.21)	0.429
	*Female*	4.35 (3.19)		2.12 (2.55)	
**Marital status**	*Married*	3.92 (3.20)	0.338	2.29 (2.97)	0.900
	*Unmarried*	3.32 (3.28)		2.36 (2.76)	
	*Elementary school and below*	3.09 (3.51)		1.58 (2.22)	
**Education**	*High school*	4.60 (3.23)	0.116	2.80 (3.27)	0.194
	*University and above*	3.62 (3.04)		2.37 (2.93)	
**Living status**	*Living with others*	3.79 (3.28)	0.681	2.29 (2.97)	0.704
	*Living alone*	3.33 (2.00)		2.67 (1.32)	
**Smoking**	*Non-smoking*	3.79 (3.24)	0.974	2.13 (2.65)	0.109
	*Quit*	3.63 (4.02)		3.75 (4.37)	
	*Current smoker*	3.67 (2.16)		2.20 (2.73)	
**BMI**	*≥25*	3.75 (3.05)	0.981	2.16 (2.59)	0.468
	*<25*	3.74 (3.26)		2.51 (3.18)	
**History of psychiatric disorder diagnoses**	*Yes*	3.2 (3.55)	0.569	1.60 (1.96)	0.426
	*No*	3.8 (3.21)		2.36 (2.95)	
**History of psychotropic medication use**	*Yes*	4.00 (4.45)	0.801	3.18 (4.45)	0.302
	*No*	3.74 (3.13)		2.24 (2.76)	
**Comorbid physical illnesses related to severe COVID-19**^a^	*Yes*	3.80 (3.12)	0.856	2.62 (3.25)	0.089
	*No*	3.70 (3.38)		1.85 (2.24)	
**Length of hospital stay (days)**	*≤15*	3.34 (3.01)	0.031*	2.05 (2.40)	0.193
	*>15*	4.51 (3.46)		2.76 (3.60)	
**ICU stay**	*Yes*	3.89 (3.29)	0.798	2.34 (3.10)	0.938
	*No*	3.73 (3.21)		2.30 (2.85)	
**Use of intubation**^b^	*Yes*	4.69 (4.31)	0.278	2.69 (3.97)	0.620
	*No*	3.68 (3.11)		2.27 (2.80)	
**CRP level**	*≥7.5*	4.06 (3.43)	0.335	2.81 (3.38)	0.090
	*<7.5*	3.55 (3.06)		1.96 (2.46)	
**Use of steroid**^c^	*Yes*	4.00 (3.28)	0.073	2.44 (2.90)	0.281
	*No*	2.84 (2.83)		1.81 (2.88)	
**Use of Remdesivir**^d^	*Yes*	3.95 (3.45)	0.422	2.67 (3.23)	0.082
	*No*	3.53 (2.92)		1.87 (2.38)	
**Use of Tocilizumab**^e^	*Yes*	3.50 (3.11)	0.405	2.40 (3.10)	0.617
	*No*	3.94 (3.30)		2.21 (2.77)	
**HADS- anxiety**	*≥8*			6.47 (4.75)	<0.001*
	*<8*			1.85 (2.22)	
**HADS- depression**	*≥8*	7.82 (4.71)	0.002*		
	*<8*	3.45 (2.87)			

Univariable comparisons were done using two-sample *t*-test and ANOVA.

*p < 0.05.

**Abbreviation:** SD: Standard Deviation; BMI: Body Mass Index; ICU: Intensive Care Unit; CRP:C- Reactive Protein.

a Comorbid physical illnesses related to severe COVID-19 infection included diabetes mellitus, hypertension, cerebrovascular disease, cardiovascular disease, pulmonary disease, and cancer.

b Use of intubation: patients receiving intubation during hospitalization.

c Use of steroid: patients receiving steroids during hospitalization.

d Use of Remdesivir: patients receiving Remdesivir during hospitalization.

e Use of Tocilizumab: patients receiving Tocilizumab during hospitalization.

### 3.3 Associations between HADS scores and demographic/clinical correlates

As presented in Table 2, the female patients, patients aged >55 years, and patients hospitalized for more than 15 days had higher anxiety scores than did the male patients, patients aged ≤55 years, and patients hospitalized for ≤15 days, respectively (Fig. 2). Table 3, Table 4 showed that in the multivariable regression analyses, female sex and HADS depression scores of ≥8 were positively associated with HADS anxiety scores in the inpatients with COVID-19; those with HADS anxiety scores of ≥8 were positively associated with HADS depression scores in the multivariable regression analysis. To further validate the results, we explored the relationship between HADS score ≥8 as the cutoff for anxiety and depression and sociodemographic and clinical variables using Poisson regression models. The results as presented in Table 5, revealed nonsignificant findings for these variables of interest.

**Table 3:** Multivariable regression for factors associated with HADS anxiety score.

Significant factors	Standard β	*P*	Factors not significant	*p*
**Gender**	0.174	0.023	**Age**	0.276
**HADS depression ≥ 8**	0.333	<0.001*	**Length of hospital stay**	0.199
			**Use of steroid**^a^	0.084

*p < 0.05.

a Use of steroid: patients receiving steroids during hospitalization.

**Table 4:** Multivariable regression for factors associated with HADS depression score.

Significant factors	Standard β	*P*	Factors not significant	*p*
**HADS anxiety ≥ 8**	0.490	<0.001*	**Gender**	0.348
			**Age**	0.461
			**Length of hospital stay**	0.549
			**CRP ≥ 7.5 mg/dl**	0.243
			**Comorbid physical illnesses related to severe COVID-19**^a^	0.113
			**Use of Remdesivir**^b^	0.595

**p < 0.05.

a Comorbid physical illnesses related to severe COVID-19 infection included diabetes mellitus, hypertension, cerebrovascular disease, cardiovascular disease, pulmonary disease, and cancer.

b Use of Remdesivir: patients receiving Remdesivir during hospitalization.

**Table 5:** Poisson regression for factors associated with HADS anxiety and depression score ≥8.

	HADS anxiety ≥8		HADS depression ≥8	
Factors	**Prevalence ratio**	***p***	**Prevalence ratio**	***p***
**Age**	0.990	0.949	0.728	0.167
**Gender**	1.125	0.460	1.196	0.352
**Marital status**	1.079	0.656	1.257	0.262
**Education**		0.716		0.384
**Smoking**		0.156		0.762
**BMI**	0.954	0.768	0.860	0.519
**Comorbid physical illnesses related to severe COVID-19**^a^	0.954	0.768	0.862	0.563
**Length of hospital stay**	0.889	0.46	0.863	0.464
**ICU stay**	0.218	0.20	0.815	0.374
**Use of intubation**^b^	0.073	0.057	0.700	0.212
**CRP level**	0.824	0.22	0.972	0.886
**Use of steroid**^c^	0.767	0.302	1.039	0.876
**Use of Remdesivir**^d^	0.440	0.235	1.039	0.876
**Use of Tocilizumab**^e^	0.909	0.585	1.040	0.836
**HADS- anxiety**			0.836	0.35
**HADS- depression**	0.889	0.46		

*p < 0.05.

Abbreviation: BMI: Body Mass Index; ICU: Intensive Care Unit; CRP:C- Reactive Protein.

a Comorbid physical illnesses related to severe COVID-19 infection included diabetes mellitus, hypertension, cerebrovascular disease, cardiovascular disease, pulmonary disease, and cancer.

b Use of intubation: patients receiving intubation during hospitalization.

c Use of steroid: patients receiving steroids during hospitalization.

d Use of Remdesivir: patients receiving Remdesivir during hospitalization.

e Use of Tocilizumab: patients receiving Tocilizumab during hospitalization.

## 4 Discussion

To our knowledge, this is the first study to specifically examine the presence of anxiety and depressive symptoms among inpatients with COVID-19 in Taiwan. In the present study, 9.9 % and 7.2 % of the inpatients with COVID-19 were likely to have anxiety and depressive symptoms, respectively, when an HADS score of ≥8 was used as a cut-off point. The female patients, those aged >55 years, and those hospitalized for >15 days had significantly higher anxiety scores than did the corresponding comparison groups.

Studies have reported varied results regarding the prevalence of anxiety and depressive symptoms in inpatients with COVID-19—19%–60 % for anxiety and 13%–80 % for depression, respectively [[15], [16], [17],^21^]. One study conducted in Cameroon using the HADS reported a considerably high prevalence of depression (81.40 %) in inpatients with COVID-19, and the prevalence of anxiety was 60.35 % [21]. Another study performed in China evaluating anxiety and depressive symptoms via the HADS reported that 34.72 % and 28.47 % of inpatients with COVID-19 experienced anxiety and depressive symptoms, respectively [22]. The lower prevalence of anxiety and depressive symptoms reported in the present study may have resulted from the culturally determined “response bias.” Cultural stoicism has been used to explain the lower prevalence of major depressive disorder (MDD) in the Taiwanese population. When a structured measurement or diagnostic interview is used among individuals who tend to repress their feelings, the “response bias” may likely lead to a lower estimate of the prevalence of emotional problems in this population [33]. For instance, prior surveys showed a lower prevalence of MDD in Taiwan, and individuals with MDD in Taiwan were found to have more lost workdays and might have higher severity of symptoms and associated functional impairments when receiving the diagnosis of MDD using the same diagnostic measure [33,34]. In addition, differences in the methods of data collection may contribute to differences in the prevalence of psychiatric symptoms. Most of the aforementioned studies have used a self-rated questionnaire to collect information regarding psychiatric symptoms, whereas in the present study, HADS scores were generated by collecting data via telephone counseling conducted by trained mental health workers, which are different from self-assessed or online application questionnaires in terms of having real interactions with individuals. Patients with COVID-19 feel lonely, anxious, or depressed during hospital isolation [11,12]. Dorman-Ilan et al. reported that increased anxiety was associated with the feeling of isolation in inpatients with COVID-19 [35]. Thus, during the telephone counseling session, the trained mental health workers may not have only evaluated the patients’ mental health condition but also emotionally supported the patients in their hospital isolation.

Consistent with the findings of previous studies, the female inpatients with COVID-19 or the patients aged >55 years had higher anxiety scores in the current analysis [[14], [15], [16],^35^]. A study conducted in China also reported that for COVID-19 patients, female sex and those aged over 50 years were associated with higher HADS anxiety scores [22]. Moreover, longer hospital stays were positively associated with HADS anxiety scores. The mean length of hospital stay in the present study was 15.73 days, which is comparable to that reported in a study conducted in Spain including 2150 inpatients with COVID-19 (mean hospital stay: 14 days) [36]. In the aforementioned study, longer hospital stays were positively associated with a new diagnosis of mood or anxiety, stress, or adjustment disorder [36]. Therefore, when inpatients with COVID-19 require hospitalization for a longer period, mental health support and the related resources should be prioritized and relocated, to provide emotional support and to reduce the prevalence of mental health problems.

In the present study, we examined the association between COVID-19 treatment regimens and psychiatric symptoms. Despite not reaching statistical significance, the use of remdesivir seems to be related to higher HADS depressive scores, whereas the use of steroids might be associated with anxiety symptoms. Although speculative, a possible explanation for these findings regarding COVID-19 treatment regimens is that medication use can make inpatients with COVID-19 feel that their illness is more severe than they believe. Greater self-perceived illness was significantly associated with a higher Patient Health Questionnaire-9 score, higher General Anxiety Disorder-7 score, and higher Insomnia Severity Index score in inpatients with COVID-19 [37]. Until now, the understanding regarding how specific medications affect the mental health of patients with COVID-19 remains limited. Future studies are warranted to elucidate the underlying mechanisms involved.

The study has several limitations. Some studies have reported that family members being diagnosed as having COVID-19 affects the mental health of inpatients; having a family member with confirmed COVID-19 was an independent risk factor for depression [15,17,22]. The death of a family member from COVID-19 was independently associated with a higher depression severity index and anxiety score [16]. Besides, lack of social support was associated with higher anxiety and depression symptoms for COVID-19 inpatients [22]. Owing to the limitations of the current study design, the aforementioned factors regarding family members and social support were not assessed. We were not able to collect information regarding diagnoses of post-traumatic stress disorder and somatization due to the retrospective nature of the study. As this study was based on data from a single hospital, the results obtained cannot be generalized to all inpatients with COVID-19 in Taiwan. Variables included in the presented model were not all fit for a normal distribution. Causal relationships among sociodemographic data, treatment regimens, and severity of psychiatric symptoms could not be established based on results from the present study.

## 5 Conclusion

We found that among the inpatients with COVID-19, 9.9 % had anxiety and 7.2 % had depressive symptoms in this study. COVID-19 inpatients with either anxiety or depression were associated with longer length of hospital stay. While demographic factors such as age and sex were shown to be associated with higher anxiety scores. Future studies are warranted to further elucidate underlying mechanisms potentially related to anxiety and depressive symptoms in patients with COVID-19.

## Ethical standards

The current study was reviewed and approved by FEMH Research Ethics Committee. The participants’ written informed consents were waived due to the retrospective design and the de-identified nature of the data in this study.

## Data availability statement

Due to the sensitive nature of data used in the current study, there are ethical or legal restrictions on sharing de-identified dataset, required by the research ethics review committee.

## Funding

This study was supported by grants from the 10.13039/501100004663Ministry of Science and Technology (MOST 109-2314-B-418 -010; and MOST 110-2314-B-418-004) and 10.13039/501100005866Far Eastern Memorial Hospital, Taiwan (FEMH-2020-C-020; FEMH-2021-C-024; FEMH-2022-C-075; and FEMH-2023-C-083).

## CRediT authorship contribution statement

**Wei-Chen Lee:** Conceptualization, Data curation, Formal analysis, Methodology, Writing – original draft. **Chun-Lin Chen:** Conceptualization, Data curation, Formal analysis, Methodology, Writing – review & editing. **Yi-Ju Pan:** Conceptualization, Data curation, Formal analysis, Methodology, Writing – review & editing.

## Declaration of competing interest

The authors declare that they have no known competing financial interests or personal relationships that could have appeared to influence the work reported in this paper.
